# Antidiabetic Activity of the Ethanol Extract of *Capparis sepiaria* L Leaves

**DOI:** 10.4103/0250-474X.43008

**Published:** 2008

**Authors:** P. Selvamani, S. Latha, K. Elayaraja, P. Suresh Babu, J. K. Gupta, T. K. Pal, L. K. Ghosh, D. J. Sen

**Affiliations:** Department of Pharmaceutical Technology, Bharathidasan Institute of Technology, Anna University, Tiruchirappalli-620 024, India; 1 Department of Pharmaceutical Technology, Jadavpur University, Kolkatta-700 032, India; 2 Shri Sarvajanik Pharmacy College, Mehsana-384 001, India

**Keywords:** *Capparis sepiaria*, streptazotocin (STZ), antidiabetic activity and Capparidaceae

## Abstract

*Capparis sepiaria* L, a profusely branched hedge plant, is used in Indian traditional medicine. *Capparis sepiaria* leaves were extracted with ethanol and concentrated to dryness. The LD_50_ value was determined as 894.43 mg/kg body weight by acute toxicity study. The ethanol extract was investigated for possible hypoglycemic effect produced by single oral administration at various dose levels 100, 200 and 300 mg/kg in the streptozotocin induced diabetic rats and compared against normal saline control and the standard glibenclamide. A maximum fall of plasma glucose level 9.40%; 13.57%; 15.25% and 18.80% was observed after 12 h of treatment when administered with ethanol extract of *Capparis sepiaria* at 100, 200 and 300 mg/kg, and glibenclamide 10 mg/kg dose, respectively. The findings from the study suggest that the *Capparis sepiaria* leaves may be prescribed as an adjunct to traditional formulation and drug treatment for controlling diabetes mellitus.

*Capparis sepiaria* L (Fam: Capparidaceae) is a profusely branched hedge plant with slender prickly shrubs, zigzag stems^1^–^2^. Traditionally, *C. sepiaria* is used as blood purifier, stomachic, tonic and appetizer. It’s flowers, leaves and roots are used in cough and toxemia and root powder is also used as a cure for the snakebite^3^. It also possesses febrifuge properties and is used to treat skin diseases, tumors, inflammation and diseases of the muscles^4^. *C. sepiaria* along with *Oxalis corniculata* and *Ricinus communis* were used for the treatment of aphthae^5^. No reports have been found concerning the pharmacological action of leaves of *Capparis sepiaria*. Therefore, the present study was undertaken to evaluate the putative anti-diabetic activity of the crude ethanol extract of the aerial parts of *Capparis sepiaria*.

Fresh leaves of *C. sepiaria* were collected form Tiruchirappalli, Tamil Nadu, during June-July and identified by Botanical Survey of India, Coimbatore where a voucher specimen (BSI/SC/5/21/04-05/TECH-7) was deposited. The plant material was dried under shade, powdered and extracted with ethanol using a Soxhlet apparatus. The resulting ethanol extract was evaporated on a water bath to give a dry extract (8.2% w/w) which was then stored in a refrigerator. Conventional protocols for detecting the presence of various phytoconstituents were employed in screening the ethanol extract of *C. sepiaria* (EECS)^6^. The presence or absence of saponins, tannins, flavonoids, alkaloids were observed.

A weighed quantity of the dried ethanol extract was reconstituted in normal saline and evaluated for pharmacological activities. Wistar rats (150-200 g) and Swiss mice (20-25 g) of either sex were used for the investigation. The animals were housed in standard environmental conditions and fed with a standard pellet diet and tap water *ad libitum*. All the pharmacological study protocols have met with the approval of the Institutional Animal Ethics Committee.

The method described by Lorke^7^ was employed for the determination of the LD_50_ and the study was approved by the animal ethics committee. Fifty Swiss mice were separated into five groups, each consisting of five males and five females, each weighing 20-25 g (n=10). They were fasted overnight and administered with the ethanol extract at the following doses: 100, 200, 400, 800 and 1000 mg/kg, i.p. Animals were observed for 24 h after drug administration. The general signs and symptoms of toxicity, intake of food, water and mortality were observed and recorded. The LD_50_ value was calculated from the square root of the product of the lowest lethal dose and highest non-lethal dose, i.e., the geometric mean of the consecutive doses for which 0 and 100% survival rate.

Wistar rats of either sex weighing 150-200 g fasted for 18 h were made hyperglycemic by a single intraperitoneal injection of streptozotocin (STZ) dissolved in 3 mM citrate buffer, pH 4.5 at a dose of 50 mg/kg body weight. After 48 h of STZ injection, rats exhibiting plasma glucose level of >250–300 mg/dl were included in the study and divided into five groups of six animals each^8^. One group served as control, which received normal saline (10 mg/kg, p.o.), second group received the standard drug glibenclamide (10 mg/kg, p.o.) and other three groups received EECS reconstituted in saline (100, 200 and 300 mg/kg, p.o.). Blood samples (0.1 ml) were collected from the tail vein of the rats at 0, 4, 8 and 12 h, respectively after oral administration. The blood sugar concentration was determined by O-toluidine method and the results were tabulated (Table 1). The percent glycemic change in the experimental animals was calculated at a time function using the following formula, % glycemic change = [initial concentration-concentration/initial concentration × 100. The data were represented as mean±SEM and statistical significance between the treated and control group was analyzed by means of students ‘t’-test; P<0.05 implies significance.

**TABLE 1:** ANTIDIABETIC ACTIVITY OF ETHANOL EXTRACT OF *CAPPARIS SEPIARIA* L. LEAVES

Treatment and dose	Plasma glucose concentration (mg %)			
	Time after treatment (h)			
	
	0 h	4 h	8 h	12 h
Control 10 ml/kg	273.50±1.11	272.56±1.05	269.33±3.80	265.83±5.20
EECS 100 mg/kg	270.26±1.02	254.83±1.25*(6.19)	253.33±2.55*(5.59)	240.83±3.65*(9.40)
EECS 200 mg/kg	265.20±2.37	252.50±3.04*(7.05)	245.83±2.53*(8.38)	230.00±1.75*(13.57)
EECS 300 mg/kg	265.00±1.63	251.66±5.47*(7.26)	240.00±3.75*(10.55)	225.00±4.62*(15.25)
Glibenclamide 10 mg/kg	265.00±2.6	242.50±2.35*(10.73)	238.33±4.85*(11.18)	215.00±3.75*(18.80)

EECS - ethanol extract of *Capparis sepiaria*. Values are expressed in mean±SEM (n=6); Numbers in parentheses denotes percentage of reduction (plasma glucose). Students‘t’ test

* P<0.05 vs. saline control

Phytochemical screening of EECS revealed the presence of alkaloids, flavonoids, steroids, glycosides, tannins and saponins. The LD_50_ of EECS was estimated to be 894.43 mg/kg in mice when administered intraperitoneally. STZ is a commonly employed compound for induction of type-1 diabetes by rapid depletion of beta cells, which leads to reduction in insulin release^9^. The effects of treatment with the EECS on blood glucose levels in STZ-induced diabetic rats are summarized in Table 1.

The crude ethanol extract exhibited antidiabetic property in STZ-induced diabetic rats as evident from the findings. At 12 h post administration, the percentage of blood glucose lowering potential was observed as 9.40%, 13.57% and 15.25% in the groups administered with EECS at a dose of 100, 200 and 300 mg/kg, respectively; while the standard glibenclamide caused 18.80% reduction of blood glucose at a dose of 10 mg/kg. The percentage increase of blood glucose in the untreated group appeared to be higher than that in the treated group. Comparison of the average values of blood glucose levels in the treated and untreated (control) groups of STZ-induced diabetic rats suggest some favorable antidiabetic effect of *C. sepiaria*. However, statistical analysis using two-tailed student’s t-test revealed that, there is a statistically valid difference between the treated and the control groups. The EECS exhibited significant (P<0.05) anti-hyperglycemic effect at 12 h post oral administration, at a dose of 200 and 300 mg/kg. The data obtained from the present study clearly confirm that the EECS tested to possess marked hypoglycemic activity on the STZ-induced diabetic rat model. From the present experimental results, it can be suggested that, the EECS exhibited dose dependent action in a similar mechanism as glibenclamide i.e., by stimulation of surviving beta cells to release more insulin^10^. It has also been described that glibenclamide and a natural hypoglycemic product were effective in moderately streptozotocin diabetic animal and ineffective in the severe diabetic rat^10^. Herbal extracts containing flavonoids and tannins were reported to demonstrate antidiabetic activity^11^. On the basis of the above evidence, it is possible that the flavonoids and tannins present in this plant may be responsible for the observed antidiabetic activity.
